# Basic Principles of RNA Interference: Nucleic Acid Types and In Vitro Intracellular Delivery Methods

**DOI:** 10.3390/mi14071321

**Published:** 2023-06-27

**Authors:** Marie Isenmann, Martin James Stoddart, Rainer Schmelzeisen, Christian Gross, Elena Della Bella, René Marcel Rothweiler

**Affiliations:** 1 Department of Oral and Maxillofacial Surgery, Faculty of Medicine, University of Freiburg, Hugstetterstrasse 55, 79106 Freiburg, Germany; 2 AO Research Institute Davos, Clavadelerstrasse 8, 7270 Davos, Switzerland

**Keywords:** RNA interference, siRNA, miRNA, shRNA, piRNA, ASO, gene silencing

## Abstract

Since its discovery in 1989, RNA interference (RNAi) has become a widely used tool for the in vitro downregulation of specific gene expression in molecular biological research. This basically involves a complementary RNA that binds a target sequence to affect its transcription or translation process. Currently, various small RNAs, such as small interfering RNA (siRNA), micro RNA (miRNA), small hairpin RNA (shRNA), and PIWI interacting RNA (piRNA), are available for application on in vitro cell culture, to regulate the cells’ gene expression by mimicking the endogenous RNAi-machinery. In addition, several biochemical, physical, and viral methods have been established to deliver these RNAs into the cell or nucleus. Since each RNA and each delivery method entail different off-target effects, limitations, and compatibilities, it is crucial to understand their basic mode of action. This review is intended to provide an overview of different nucleic acids and delivery methods for planning, interpreting, and troubleshooting of RNAi experiments.

## 1. Introduction

In 1928, an astonishing observation was made during experiments with viruses on tobacco plants by Wingard et al., who observed that only the first leaves infected with the ringspot virus developed the full virus disease, while the upper leaves showed a miraculous recovery and resistance. Wingard was not able to explain this on a molecular biological level, but this recovery phenomenon formed the starting point for the discovery of the mechanism of RNA interference (RNAi).

More than 50 years later, Izant et al. showed that injection of complementary transcripts into mouse cells reduced the expression of specific genes [1]. The concept of “pathogen-derived resistance” was developed by Abel et al. in 1986, whereby defective expression of a pathogen’s gene product resulted in protection against that pathogen. However, this concept was still based on the assumption that the interaction occurred at the level of gene products (protein complexes) [2]. In 1989, Powell et al. studied the effect of antisense and satellite RNA and found that nucleic acid interaction was responsible for this protective effect [3].

The phenomenon of complementary nucleic acids inhibiting each other is conserved in most eukaryotes and is an indispensable part of the physiology of many organisms [4]. In plants, it balances the organism’s efforts between pathogen defense and growth [5]. As shown as early as 1928, RNA interference plays a central role in virus defense, as the double stranded RNA (dsRNA) triggers the plant RNAi system to silence complementary genes, thereby generating immunity [6,7]. In mammalian organisms, endogenous RNAi systems are important elements for the control of development, fate, and death of cells in various physiological and pathological states.

In experiments studying the effects of gene expression, targeted knockout has become an indispensable procedure to gain new insights. For a long time, culturing knockout organisms was the gold standard for this purpose, limited by the enormous time and costs, ethical concerns, and limited analysis of individual tissue or cells. RNA interference has overcome these obstacles, and today, there are numerous companies offering a wide range of artificial nucleotide acids for RNAi as well as various carrier systems for intracellular delivery.

In this review, we aim to provide an overview of the basic biological principles of RNA interference, available nucleotide acids, and in vitro delivery systems for use in gene expression experiments.

## 2. Principles of RNA Interference

The term RNA interference describes the principle of reducing the expression of a particular gene by complementary short RNAs. In general, this effect can be induced after transcription by mRNA cleavage or translation repression and at the transcriptional level by transcriptional silencing [8].

The process starts with RNA-dependent RNA polymerases (RdRPs) that generate long dsRNA from single stranded RNA templates [9,10]. The long dsRNA is transferred by endocytosis to the cytosol and processed by endoribonuclease Dicer or Tar-RNA-binding protein (TRBP) [11]. The resulting nucleic acid is called siRNA [12,13].

In the following steps, the nucleic acid is loaded onto the RNA-induced silencing complex, or RISC. This multiprotein RNA complex plays an essential role in the RNA silencing process. Its function is mainly based on Argonaute proteins that occur in subclades for miRNA, siRNA, or piRNA processes and contain specific domains [8]. The *N*-domain of Argonautes unwinds the single strand of RNA, while the PAZ-domain binds the 3′-overhang [14,15,16]. The MID-domain binds the 5′ end, while the PIWI-domain can cleave the target sequence [17,18]. In this process, the RISC selects one strand as the guide or antisense strand, while the complementary passenger strand is degraded. This strand selection is influenced by thermodynamic stability and nucleobase of the 5′ end [15]. The entire process is dependent on HSP90 proteins that keep the Argonautes in the correct conformation [19].

After the process of RISC loading, the attached siRNA is 20–27 nt long and directs the RISC to its complementary target sequence, either RNA or DNA. Subsequently, DNA methylation or chromatin modification inhibits the transcription of DNA (TGS), and mRNA cleavage or translation inhibition affects the posttranscriptional processes (PTGS) [20].

**Figure 1:**
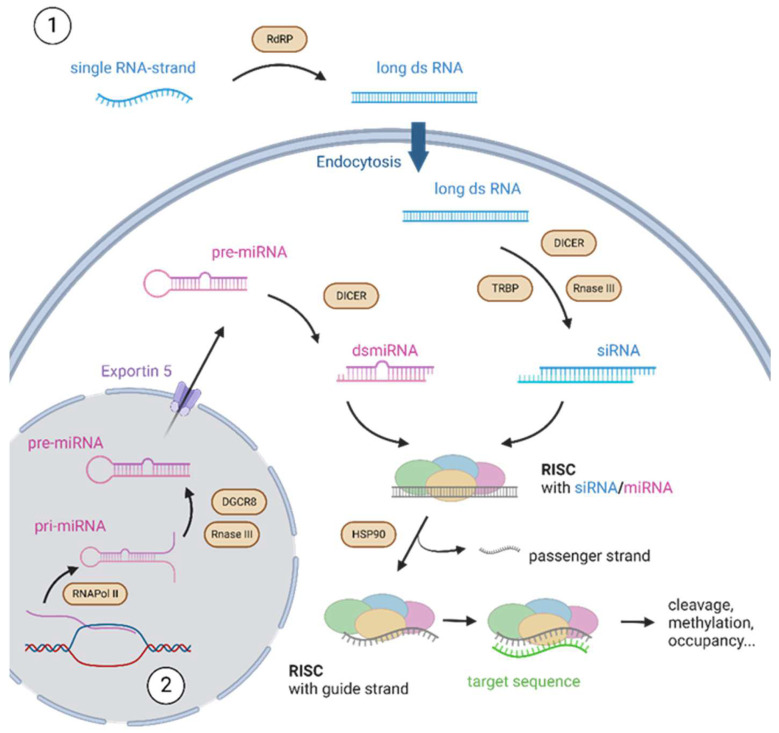
Biological principle of RNA interference: 1. siRNA-pathway: RdRPs generate long dsRNA from single-stranded RNA templates, that are taken up by endocytosis and processed into siRNA by Dicer or TRBP which is loaded onto RISC. 2. miRNA-pathway: RNAPol II transcribes pri-miRNA, which is processed by RNAse III and DGCR8 protein to pre-miRNA. Pre-miRNA is exported by exportin 5 and processed by Dicer to dsmiRNA, which is loaded onto RISC. The passenger strand is degraded, and the guide strand can bind the target sequence and alter gene expression, by cleavage, methylation, translation inhibition, etc.

MiRNAs have a similar yet different origin, function, and purpose. The polynucleotides are approximately 18–26 nt long, single stranded, and in a stem-loop structure. Their production begins with the transcription of specific genes by RNA polymerase II [21]. The resulting pri-miRNA is capped and polyadenylated and further processed by the Drosha (RNAse III) and DGCR8 protein [22]. This pre-miRNA is hairpin-structured and exported from the nucleus by exportin 5 [23]. Subsequently, Dicer cleavage forms a 21 nt ds miRNA, which is loaded onto RISC similar to siRNAs [24]. However, in some cases both strands of miRNA (passenger and guide strand) bind a target sequence and affect gene expression [25]. Unlike siRNAs, miRNAs are often only partially complementary to their target sequence [26]. More specifically, they target 3′ UTR regions of mRNAs, with nucleotides 2–8 being in most cases fully complementary and referred to as the “seed region” with canonical binding, while the remaining part is only partially complementary [27]. In miRNAs, the target sequence-RISC interaction usually does not lead to cleavage by Dicer, but to recruitment of the GW182 protein [28]. Through the interaction with the cytoplasmic poly(A)-binding protein PABPC, GW182 induces both translation repression and mRNA deadenylation, the latter followed by 5′-cap removal and mRNA degradation by exoribonucleases [29] (Figure 1).

## 3. Nucleic Acids in RNAi

### 3.1. siRNA

siRNAs play an important role in gene expression silencing for research and potential therapeutic use. siRNAs are less likely than longer nucleotides to cause immune stimulation. They can be transported between different tissues in some species and are very precise due to their full complementarity. Overall, siRNAs are highly efficient tools for in vitro experiments and pose fewer problems than other interfering RNAs [30,31,32].

Nevertheless, many side effects have been observed with the widespread use of siRNA, which can currently be explained by three mechanisms:

#### 3.1.1. miRNA-like Off-Target Effects

Off-target effects are often caused by siRNA binding to non-target genes that have partial complementarity to the 5′ end of their guide strand [33,34]. The exogenous siRNA essentially takes over the function of an endogenous miRNA, causing unintended effects on cell growth or altering other experimental outcomes [35,36]. This phenomenon is referred to as the “miRNA-like off-target effect”. Since this principle is part of cell physiology, it cannot be completely eliminated; however, there are strategies to reduce the likelihood that this phenomenon occurs.

One option is improving the siRNA sequence design by analyzing the whole genome of target cells and avoiding sequences that could induce miRNA-like effects [37,38].

Second, chemical modification of siRNA reduces off-target-effects by destabilizing the two strands. Since miRNA-like bonds are shorter than the intended siRNA target bond, they are more affected by this destabilization. This modification can be either 2′-*O*-methylation or locked nucleic acid (LNA) incorporation, which is particularly effective at position 2 of the 5′ end [35,38].

As a third option, lower siRNA-concentration has been shown to reduce these off-target effects [39,40,41]. Since a mere reduction in siRNA-concentration also reduces the target effects [40], the method of siRNA-pooling was developed. The method uses a pool of siRNA sequences that all target the same gene but bind at different sites. There are several strategies for creating such a pool. The least complex pools are produced by combining just a small number of siRNAs that share the same target gene (e.g., smart pools with four different siRNAs). Their dilution effect, which should reduce the miRNA-like off-target effects, is relatively low. This dilution effect is increased with endoribonuclease-produced siRNA pools (esiRNA), produced by digestion of dsRNA using RNAse III [42] which results in hundreds of different siRNAs [43]. Third, so-called siPools with about 30 different sequences, in contrast to esiRNA, are designed in vitro and therefore are well-defined but also more costly to produce. They eliminate sequence-specific off-target effects such as esiRNA, while it is much easier to control and understand their effects on cells [43].

#### 3.1.2. Immunostimulatory Response

SiRNA avoids some immunostimulatory response due to their size of less than 30 nt. Nevertheless, they can still trigger an immune response [44]. The immune activation is concentration-dependent and detectable with each siRNA application [45]. Besides dsRNA, carriers can also be immunostimulatory triggers. Endosomal transfection systems have been shown to be much more likely to cause immune stimulation because the endosome contains many immune-activating receptors [44,46,47]. Many of the effects are also sequence-dependent, which reduces the informative value of nonbinding sequences as negative controls but can be reduced by avoiding immunostimulatory motifs [48]. Cell type also influences appearance and extent of the immune activation [49].

In general, there are three distinct signaling pathways for siRNA-induced immune stimulation.

First, dsRNA can bind dsRNA recognition proteins, which triggers antiviral responses and causes upregulation of Interferon (IFN) and other antiviral proteins. IFN activates IFN-stimulated genes (ISG) such as PKR that inhibit viral replication and protein synthesis [50]. Second, dsRNA activates oligoadenylate synthetases (OAS). They convert ATP to oligoadenylates, thereby activating RNAse L, which is capable of degrading intracellular single-stranded RNA [51]. Third, dsRNA binds to Toll-like receptors (TLR) and to transcription factor IRF3, leading to the induction of IFN, TNF-alpha, and IL6 [44].

Activation of the cell immune system can have many complex effects on the target cells and the experimental outcome. Chemical modification of siRNA, such as 2′-*O*-methylation or locked nucleic acid incorporation, is one approach to reduce this problem [52].

#### 3.1.3. Saturation of Endogenous RNA Interference

Exogenous siRNA can affect the endogenous RNA interference machinery of cells. High siRNA concentrations lead to intense siRNA-RISC loading, which may reduce the ability to generate RISC for miRNA-induced silencing. The reduced miRNA suppression may lead to undesired gene expression, thereby affecting cell phenotype [53].

Considering all these challenges, choosing an appropriate siRNA sequence is not trivial. This sequence should not only be complementary to the target gene but also very specific and preferably not affecting other genes or signaling pathways in the cell. Sequence design is mainly done in silico applying several rules. A low G/U content is preferred as this reduces immunostimulation [54], as is low internal stability at the 5′ end of antisense strands to facilitate RISC entry. Stable internal repeats are avoided since they cause internal folding that interferes with target binding [55].

### 3.2. miRNA

MiRNAs, short for micro RNAs, are 21 to 25 nt long, occur ubiquitously in eukaryotic cells, and form a stem-loop-structure [56]. They not only inhibit gene expression but can also induce transcription by mRNA-promoter binding [57]. Most miRNAs are formed by modifying specific DNA-transcripts (pre-miRNAs) that are exported from the nucleus and processed by Dicer enzymes. In addition, there are other, non-canonical pathways for miRNA production. These include so-called “mirtrons”, spliced introns of mRNA, fully Dicer-independent miRNA which is derived from endogenous shRNA processed by Drosha and cleaved by hAgo2 (Human Protein argonaute-2) or m7g (7-methylguanosine)-capped pre-miRNA that can be exported to the cytoplasm without Drosha cleavage [58,59,60]. In many cases, multiple miRNAs are transcribed as one long transcript (cluster) that is subsequently cleaved. These “miRNA families” usually bind similar seed regions [61].

Unlike other small RNAs, miRNAs are able to move between different compartments of an organism and can therefore be detected in extracellular fluids [62,63].

Currently, miRNAs have gained importance due to their expression in various diseases, especially cancer [64].

However, miRNAs are less suitable for in vitro analysis of gene expression and for experiments that require precise gene silencing. Their complementarity is not perfect, resulting in unstable and non-specific mRNA binding that can even be toxic [26]. The main benchside application of miRNA is to analyze and validate their expected effects on gene expression and phenotype of cells to decide on further investigations and possible therapeutic applications [65]. To this end, cell cultures are transfected with a miRNA mimic and a scramble sequence [66]. However, in these experiments, miRNAs show many side effects, such as causing interferon response, strand bias, or unspecific binding to non-target sites [67,68,69]. For this reason, miRNA inhibitors are the preferred approach for miRNA validation, especially miRNA sponges. MiRNA sponges are plasmids that contain many miRNA binding sites [70]. To avoid RNAse H activity, their sequences are not perfectly complementary to miRNAs. To avoid unintended binding, their design is quite complex, and they are mostly planned by using webtools such as miRNAsong, whereas also engineered circular RNA (circRNA) with miRNA-sponging function may be used [71,72,73].

### 3.3. shRNA

Short hairpin RNAs (shRNAs) are RNA sequences that form a tight hairpin based on their sequence consisting of a target specific part, a spacer, and a reverse complement of the target sequence [74].

To achieve more stable knockdown experiments, researchers have been inspired by the design of endogenous pre-miRNA in the development of shRNAs [75,76].

Usually, shRNA sequences are introduced into the cell by vectors (e.g., plasmids) and must be transcribed in the nucleus to obtain the hairpin-structured shRNA. Based on their transcription pathway, current shRNAs can be divided into first and second generation.

The first generation of shRNAs uses RNA polymerase III promoters in their vectors, in most cases the U6 and H1 promoter [77,78,79]. Transcription produces stem-loop-structured, pre-miRNA-like shRNAs in the cell. These shRNAs can be processed into more potent RNA interference nucleotides than those provided by endogenous mechanisms [80].

However, first generation shRNAs cause many off-target effects that lead to toxicity and disruption of endogenous miRNA [81,82,83].

Second-generation shRNAs mimic pri-miRNAs, a preform of pre-miRNAs that requires an additional processing step [76,84]. Their gene template is transcribed by RNA polymerase II. This transcription process involves capping and poly-A tailing [84,85]. In comparison to the first generation, this approach is more adaptable and offers the possibility of transferring entire shRNA clusters [86,87]. However, second-generation shRNAs are less well understood and more complex.

After transcription, shRNAs are processed into siRNA. This is achieved with the help of the cell’s endogenous RNAi-processing machinery. For the shRNAs to be recognized and processed by the endogenous pathways, specific signals are required in the shRNA. Since these design requirements are quite complex, endogenous miRNAs are currently used as templates for the design of shRNAs [88]. Another challenge is that cleavage sites for the same shRNA sequence have been shown to be inconsistent. Rules for length and loop position may mitigate this disturbance [89,90].

RISC loading of the resulting siRNA can be improved by aiming for hAgo2 cleavage-dependent RISC formation. Strand selection is improved by designing the 5′ end of the guide strand to be less stable than the passengers one [91,92]. For ideal target sequence binding, imperfect complementarity has been shown to result in a weaker outcome and more off-target effects [93]. Perfect matches, whereas complementarity at the 3′ end is negligible, result in more efficient hAgo2-dependent cleavage of the target [94].

Overall, shRNA systems have many advantages over siRNA. Their effect on cell gene expression lasts much longer because the vector often remains in the cell and is transcribed more than once [75]. Moreover, controllable vectors can be designed by inserting selection markers or inducible elements into the sequence and its promoter.

Nevertheless, the entire shRNA system is very complex and still not well understood. Identical shRNA sequences are processed differently in different cell lines, causing miRNA-like off-target effects and immune stimulation, that cannot yet be avoided by improved shRNA design [48,95,96]. Furthermore, shRNA must be transcribed in the nucleus, requiring vectors with precise nuclear delivery [74]. Not least, shRNA utilizes many parts of the cell’s endogenous RNAi system, which can easily lead to saturation of, e.g., exportin 5 or Argonaute proteins and thus severely disrupt the cells’ gene expression regulation [97,98].

Currently, shRNAs are widely used to transduce cells for efficient gene knockdown. They can enable mass production of siRNA in vitro, and their potential future role in treatment of viral diseases should not be underestimated due to numerous ongoing research and trials in different phases [86,99].

### 3.4. piRNA

PIWI-interacting RNAs (piRNAs) are 21–35 nt long single stranded nucleic acids that carry a 2′-*O*-methylation at their 3′ end, uridine as a terminal base at the 5′ end, or adenosine at the tenth position [100,101,102]. They do not share a specific common secondary structure [100].

PiRNAs were first identified in animal germ cells. They were found to be produced in a Dicer-independent manner, copied from non-coding genomic regions with repeats, and are an important player in posttranscriptional regulation, particularly in protecting germline cells from transposable elements (TE) [103]. PiRNAs have also been detected in somatic cells, where they are required for epigenetic regulation through methylation, transposon silencing, and chromatin modification. Their importance is particularly evident in various malignant pathogenesis pathways [104].

PiRNAs interact with PIWI proteins. PIWI proteins represent a subfamily of Argonautes and therefore play an important role in the formation and function of RISC. In this context, PIWI proteins have an endonuclease function and can cleave RNAs [105].

PiRNAs affect cell gene expression through various mechanisms. In transcriptional gene silencing (TGS), piRNA/PIWI protein complexes bind the target gene, methylate DNA, and modify histones [106,107,108]. In post-transcriptional gene silencing (PTGS) piRNAs act similarly to miRNAs and form a piRISC on mRNAs to prevent their translation [109,110]. Furthermore, piRNA/PIWI protein complexes modify posttranslational processes (PTM) by interacting with transcription factors, leading to their posttranscriptional phosphorylation [111,112].

Since piRNAs bind nonspecifically to different targets and their effects in cells are not yet well predicted, they are currently not used for gene expression experiments. Nevertheless, their role in controlling Tes could provide an approach for therapy in cancer or other diseases [113].

### 3.5. ASO as an Alternative to RNAi

The use of Antisense Oligonucleotides (ASOs) is an alternative approach to RNAi for the regulation of gene expression. Considering the common goals and shared challenges of RNAi and antisense approaches, ASOs are herein discussed.

ASOs are short, synthetic, single-stranded oligonucleotides (both DNA- and RNA-based) with antisense function, and they downregulate gene expression via different mechanisms [114]. Because of a DNA:RNA heteroduplex formation, some induce Rnase H-mediated target cleavage [115]. Others induce cleavage by hAgo2 and other Argonaute proteins. In addition, there are ASOs that only occupy their target, thereby either preventing translation and causing cleavage through the resulting arrest or promoting translation through altered splicing [116].

When first developed, ASOs were found to be toxic, rapidly degradable, and difficult to transfer through membranes due to their negative charge [117]. Today, multiple ASO modifications are established to overcome these obstacles [118,119].

Compared to nucleic acids for RNAi, ASOs have been shown to be more flexible. They comprise both hydrophobic and hydrophilic parts, making them amphiphilic [120]. Interestingly, discoveries in siRNA design have improved ASO development and vice versa [121]. ASOs can cross the cell membrane in different ways. Most ASOs are modified with phosphorothioates (PS-ASOs), which allow them to bind surface proteins and enter the cell through endocytosis [122]. After passive diffusion through nuclear pores, ASOs can bind their target sequence and initiate various pathways [123].

As mentioned previously, the most common ASO modification today is PS-ASO, in which the phosphodiester in the backbone is replaced by a phosphorothioate at one or more sites [120]. This modification increases the distance between the charged parts, making the molecule more lipophilic and thus facilitating protein binding [124].

Modification of 2′-C of ribose increases stability, target affinity, avoids DNA:RNA heteroduplex formation, but can also trigger inflammatory processes [116,119,123]. When RNAse H degradation is intended, 2′ modifications in the target-binding core should be avoided, only the extremities can carry modifications to increase stability (“gapmer” structure) [125]. In many cases, the core of this gapmer structure contains deoxynucleotides, with RNA flanking regions. These chimeric DNA-RNA molecules enable the formation of DNA:RNA-duplexes with the target RNA, which are well recognized by Rnase H [123].

For specific drug delivery, ASO can carry specific conjugates. For example, N-Acetylgalactosamine (GalNAc) bound to PS-ASO enhances delivery to hepatocytes, while glucagon-like peptide 1 (GLP-1)-PS-ASOs are specifically delivered to the pancreatic beta cells [123,126].

Challenges in using ASOs for in vitro knockdown include high nonspecific signals by scramble sequences, no significant knockdown, and viability reduction [127,128]. As ASO design is complicated, most researchers purchase them from manufacturers. Lacking knowledge of the exact sequence or chemistry, it is much more difficult to interpret nonspecific signals and optimize design [129,130,131]. Nevertheless, there are already approaches and studies using ASOs in therapeutic contexts to treat viral diseases, genetic alterations, cancer, chronic inflammation, and COVID-19 [130,132,133].

## 4. Intracellular Delivery

### 4.1. Biochemical Methods

#### 4.1.1. Lipid-Based Delivery

The first established lipid-based delivery method was lipofection (or lipoplex-based delivery), in which nucleic acids, lipids and polymers form complexes [134]. These complexes are mainly introduced into the cell by endocytosis, while also fusion to the membrane occurs in some cases [135].

The cationic lipids of lipoplexes interact with and neutralize negatively charged nucleic acids [136]. They contain a positively charged polar head, a hydrophobic tail, and a linker bond [137]. The type, length, and orientation of linkers have a critical impact on the efficiency, toxicity, stability and biodegradability of lipoplexes [138]. In addition, linkers can be designed to be environmentally sensitive and can be altered by pH, oxidation, or enzymes [139,140]. The most widely used and best characterized cationic lipid is the ether-linked 1,2-dioleoyl-3-trimethylammonium-propane (DOTAP) [141].

The neutral lipids contain phosphatidylethanolamines, phosphatidylcholines, or cholesterol and very often 1,2-dioleoyl-3-glycero-phosphatidylethanolamine (DOPE) is used. They decrease cytotoxicity and increase transfection efficiency [142,143].

However, lipofection shows to have several side effects and disadvantages.

The delivery of cationic lipids depends mostly on the cellular endocytosis system, whose function varies between different cell types and is very sensitive to factors that disrupt these endocytic pathways [144,145,146] Furthermore, lipofection reduces cell viability, which is mainly caused by the cytotoxicity of their headgroups [147] but also by stimulating pro-inflammatory pathways through binding of pattern recognition factors (PPRs) in endosomes [145,148,149,150,151]. Beyond that, lipofection shows relatively slow and weak effects caused by the high lysosomal degradation during endosomal delivery [152]. 

An approach to address these issues are multi-component lipoplexes, that increase efficiency 10–100-fold by destabilizing the endosome and adjusting pH through polymers [153,154,155,156]. Another possibility is the protection of nucleic acids by albumin, chitosan, or protamine. In some cases chloroquine is added to improve endosomal release [157].

To bypass endocytosis, fusogenic liposomes were developed as another lipid-based delivery agent [158]. Here DOPE, DOTAP, and an aromatic molecule are used to create a cationic liposome that fuses directly with the negatively charged cell membrane without requiring interaction with a protein [159,160,161]. This is achieved by electrostatic interactions of a delocalized π-electron system [158,162]. As a result, nucleic acids are delivered directly to the cytosol [163].

Neutral lipids can be used as a control element, as smaller head groups increase fusion efficiency [162]. However, the ratios need to be optimized as neutral lipids on the one hand reduce toxicity, but on the other hand can disrupt the interaction between the positive liposome and the negative cell membrane [164]. Overall, fusogenic liposomes are more efficient, cause less cell death and achieve much faster effects than lipoplex-based systems and therefore represent an attractive alternative for in vitro gene expression experiments [145].

Especially in vivo, so-called lipid nanoparticles (LNPs) are used. Unlike standard lipoplexes, they can carry ionizable lipids instead of cationic lipids, which are pH sensitive and can adjust their electrical charge to the environment [165]. In addition, lipids can be modified with PEG residues to be exposed on the surface of the liposome, preventing serum protein uptake, phagocytosis, and aggregation, and can be functionalized to bind specific targets, while often impeding cellular uptake and endosomal release [166].

#### 4.1.2. Polycationic Polymers

Another approach for oligonucleotide delivery is represented by the use of polycationic polymers that form polyplexes with negatively charged nucleic acids through electrostatic interaction to facilitate membrane passage and improve stability [167]. Commonly used polycationic polymers include polyethylenimine (PEI), polyaminoethyl methacrylate, and dendrimers [168]. Polyplexes can be modified to allow active and passive targeting, stimulation of endosomal release, and encapsulation of other drugs [168,169]. Despite numerous modifications and developments in polymer technology, they still have low biodegradability, which often leads to cytotoxicity and limits their application [170]. DNA-inspired nucleic acid vehicles may solve this problem [171].

### 4.2. Physical Methods

#### 4.2.1. Electroporation

**Figure 2:**
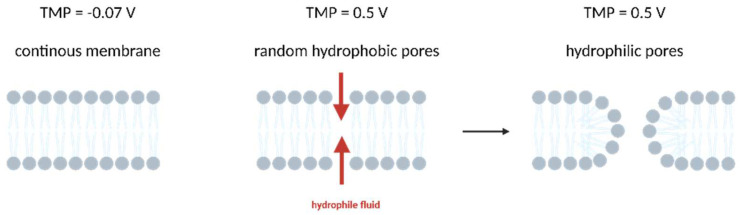
Simplified principle of electroporation: Higher TMP causes the formation of random hydrophobic pores. Liquid penetrates, making it more stable for lipids to rotate and form hydrophilic pores.

Delivery by electroporation is based on the principle that an electric field applied to a cell increases its membrane permeability. This is achieved by raising the transmembrane potential (TMP) above a certain threshold. For example, in eukaryotic cells normal TMP is at −0.07 V and the threshold at which permeability is increased is around 0.2–0.5 V [31] (Figure 2).

In this process, higher TMP raises the energy level of the membrane, causing formation of random hydrophobic pores. With TMP staying elevated, liquid enters the pores, lipids turn around and form hydrophilic pores, which is called reversible electroporation (RE) [172]. If the TMP is raised above a threshold (around 1 V), cells cannot restore a closed membrane anymore, which is called irreversible electroporation (IRE) [173]

The TMP, which is critical for the efficiency of transmission and cell viability, is determined by several parameters such as membrane diameter, cell shape and radius, the applied electric field, and the angle of the field to the cell [174]. An important factor is also the conductivity of extracellular fluid, membrane, and cytoplasm, which changes dynamically during electroporation due to ion flow [175].

The pulse frequency has an enormous influence on efficiency. In most cases, low frequencies of 1–10 Hz are chosen [176]. They are particularly suitable for longer pulses (around 100 µs) [177]. Higher frequencies may cause side effects especially in vivo [178]. However, for nanosecond pulses, higher frequencies increase efficiency [179]. Very high frequency pulses can accumulate in cells and reduce the threshold of energy required for RE [180,181]. In contrast, very low frequencies (0.1–1 Hz) can increase efficiency by electrosensitization of the membrane [182,183].

There are numerous different electroporation systems used in experimental research. Based on their size, they can be divided in major 3 groups: macro-, micro-, and nanoscale electroporation.

During macroscale electroporation, also called bulk or cuvette electroporation, multiple cells are treated at once in chambers with a diameter of at least 1 mm, providing a straightforward, inexpensive, and high-throughput transfection method [184].

Microscale electroporation is performed in chambers or channels with a diameter of micrometers. It offers many advantages over the bulk approach: smaller electrodes and lower voltages are required, therefore being better at maintaining cell viability; as surface-to-volume ratio of cells increases, there is less heat dissipation; the possibility of real-time monitoring; electrode positions can be adjusted to allow electroporation of individual cells while maintaining high-throughput through flow devices [185]. Microscale systems can have parallel or transverse electrodes, contain channels of varying width for locally enhanced electric fields, or specialized microfluids that enable droplet-based electroporation [186,187,188].

In nanoscale electroporation, charged fluids pass nanostructures (nanochannels, nanopores or nanostraws). This allows electric fields to be applied very precisely to specific membrane regions of a cell [189,190,191]. Nanofountains even exhibit a gun-like structure by applying an electric field generated with an atomic force microscope through a microcatheter with an opening of less than 1 µm [192]. While the precision of nanoscale electroporation cannot be surpassed by other systems, nanoscale electroporation is very complex and expensive to establish and has low throughput.

Overall, electroporation is a relatively inexpensive and safe approach for intracellular delivery. It is feasible for many cell types, especially primary cells where viral transduction is often insufficient [193,194,195,196,197,198,199]. However, the system configuration to achieve an appropriate TMP is challenging, because several factors need to be considered and high TMPs can reduce viability through IRE, while when TMPs are too low, the applied energy is used for heat dissipation, electrophoresis and electrolysis [173,174,175,200,201].

#### 4.2.2. Sonoporation

During sonoporation, acoustic waves are applied to cells or the fluid surrounding them to disrupt the cell membrane [202]. Most sonoporation systems rely on bubbles in the surrounding fluid (bubble-based), whereas newer approaches can disrupt the membrane without bubbles (non-bubble-based).

For bubble-based sonoporation, 3 main mechanisms are currently in use.

“Inertial cavitation” uses the jet flow generated by the bursting of bubbles due to sound waves. This jet flow leads to rupture of the cell membrane, and the fluid stream generated by the collapse also leads to membrane perforation [203,204]. However, irreversible pores lead to cell death and unstable byproducts, such as temperature dissipation and reactive oxygen species, which decrease viability [203,205,206].

“Stable cavitation” uses the shear stress generated by the fluid stream of oscillating bubbles to disrupt membranes [207]. The approach has fewer side effects than inertial cavitation, and the bubbles can also adhere directly to the cell membrane and open micropores [208,209,210]. However, this method requires precise bubble size and bubble-to-cell distance, which is often difficult to maintain even under experimental conditions [209,211].

“Acoustic radiation force” as a third mechanism pushes bubbles through the cell membrane, creating holes in it [212,213]. Factors such as bubble size, acoustic impedance, and acoustic energy density must be adjusted to achieve satisfactory results [214].

The challenges of bubble-based sonoporation are the need for a contrast agent, a specific bubble distance and bubble-to-cell ratio [209,211,215].

In non-bubble-based mechanisms, three main forces are applied to the cell: acoustic radiation force, shear force due to acoustic streaming and energy applied by an adherent substance stimulated by acoustic waves [216,217,218]. These forces stress the cell membrane, leading to pore formation. The radiation force is used to push the cells to a pressure node where they can be observed, or to push them through constricting nozzles or against walls to increase membrane stress [219,220,221]. Cells attached to acoustically stimulated substrates are directly exposed by their attachment [222]. High frequency acoustic waves as concentrated acoustic radiation can precisely target one single cell [223,224]. Hyper-frequency acoustic waves or focused transducers can even achieve membrane disruption by the stream of acoustic waves [225,226].

In summary, sonoporation is a promising tool for intracellular delivery that is suitable for various cell types and cargoes. It can be combined with other delivery methods [227,228]. Nevertheless, there are still many challenges: thermal dissipation can affect cell viability, reactive oxygen species can cause apoptosis and necrosis, and genotoxicity has also been observed [229,230,231,232].

#### 4.2.3. Microinjection

The oldest method to transfer genetic material into a cell is microinjection. Using a glass pipette with a diameter of 0.5–15 µm, fluids can be injected into floating and adherent cells [233]. This allows for targeted delivery into single cells, such as zygotes, to generate transgenic organisms [234]. However, this method has a particularly low throughput and requires an experienced researcher for cell holding, injection site selection, and volume [235]. Automated microinjection systems are currently being developed to address this challenge [235].

### 4.3. Viral Transduction

In 1967, it was discovered that adenoviruses can transiently regulate the gene expression of a cell [236]. The adenovirus genome contains so-called Early genes (E genes), that control the viral life cycle. Of these, E1A is required to initiate viral replication, while E3 does not play a critical role for viral survival or replication [237].

**Figure 3:**
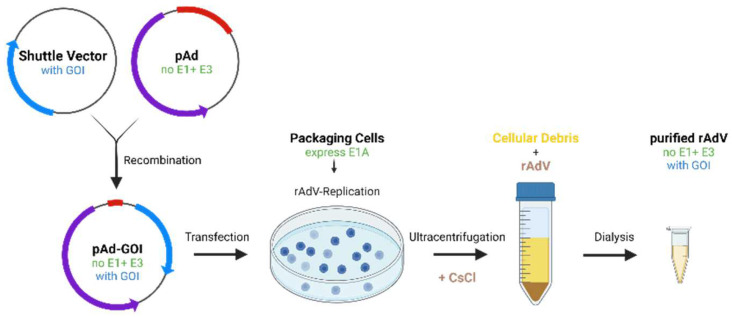
Production of recombinant adenoviruses (rAdV): A shuttle vector containing the GOI is recombined with a plasmid containing adenoviral genes but lacking E1 and E3 (pAd). The resulting pAd-GOI is transfected into packaging cells that express E1A and allow replication of rAdV. Ultracentrifugation with CsCl (cesium chloride) is used to separate rAdV from cellular debris and finally dialyzed to obtain purified rAdV.

Adenoviruses used for intracellular delivery include replication defective and conditionally replicating adenoviruses. Replication defective adenoviral vectors lack E1 and E3. Therefore, they cannot replicate but provide space for insertion of external genes [238]. They are commonly used for gene delivery in in vitro research. For construction, the gene of interest (GOI) is cloned into a plasmid vector. The final plasmids contain at least the GOI (usually shRNA in case of RNAi) in an open reading frame (ORF), a promoter and a marker gene. The adenovirus is then transfected into packaging cells that express E1A and allow adenoviral reproduction [239,240]. The replicated adenovirus, particles lacking DNA and cellular debris are separated by ultracentrifugation so that the final adenovirus contains the GOI and lacks E1 and E3, preventing it from replicating in humans. Adenoviral vectors release their genome into the nucleus, where it is not integrated into the genome but remains in the episomal state for transcription, is retained much longer than non-virally delivered nucleic acids, but considerably reduced by cell division [241,242] (Figure 3).

Conditionally replicating adenoviruses (CRA) have the E1A promoter replaced by a cancer-specific one [243]. This modification restricts viral replication to cancer cells, while benign cells are unaffected. Nevertheless, its clinical application has not been successful so far. Target specific CRAs carry promoters that depend on several factors, and viral replication is possible only when these factors are present [244].

Adeno-associated viruses (AAV are non-enveloped, single-stranded DNA viruses belonging to the Parvoviridae family [245]. Because they have low pathogenicity and immunotoxicity, a high safety profile in clinical trials, long-lasting transgene expression, and a simple genome that is easy to be modified, AAV are promising candidates for in vivo drug delivery [245,246,247]. However, AAV are dependent parvoviruses as their replication is dependent on other viruses [248]. Despite intensive research on stable production lines in recent decades, the production of high quantities of AAV is very time-consuming and costly [248]. In addition, AAV can cause damage to insertion sites and have limited capacity for transgene cargo [249,250]. Overall, viral delivery is still the most efficient and durable method for gene transfer into most cell types [251]. Nevertheless, its price and especially its higher risk profile for insertional mutagenesis and immune responses dampen enthusiasm about its use and potential, especially for in vivo therapies [252].

Another common virus family for gene transfer are lentiviruses. In lentiviruses, genes encoding viral structural proteins can be replaced by GOIs, and unlike adenoviruses, they integrate their genes into the genome of infected cells [253,254]. For this reason, modulation of gene expression with lentiviruses is exceptionally long and stable, but also entails more oncogenic risks depending on the insertion site. Lentiviruses are well suited for gene transfer as they elicit little immune response while inducing stable transgene expression [255].

## 5. Conclusions

Numerous discoveries in the field of RNA interference and intracellular delivery have been reported in the past few decades. Today, researchers can choose from a vast array of methods to perform their gene expression experiments. However, knowledge of background processes, pitfalls, and compatibilities with cells and cargo is indispensable for appropriate method selection, correct application, and meaningful interpretation. For this reason, the review provides an overview and orientation for all those approaching RNA interference and relative in vitro application.

## Data Availability

All data can be requested from the corresponding author.

## Abbreviations

**Abbreviation**	**Definition**
hAgo2	Human Protein argonaute-2
ASO	Antisense Oligonucleotides
ATP	Adenosine triphosphate
COVID-19	Coronavirus disease 2019
CRA	Conditionally replicating adenovirus
CsCl	Cesium chloride
DGCR8 protein	DiGeorge syndrome critical region 8 protein
DOPE	1,2-dioleoyl-3-glycero-phosphatidylethanolamine
DOTAP	1,2-dioleoyl-3-trimethylammonium-propane
dsDNA	Double stranded DNA
E1–4	Early-transcribed regions 1–4 in adenoviruses
esiRNA	Endoribonuclease-produced siRNA pools
GalNAc	*N*-Acetylgalactosamine
GLP-1	glucagon-like peptide 1
GOI	gene of interest
GW182	Protein Gawky
HSP90	Heat shock protein 90
IFN	Interferon
IL6	Interleukin-6 gene
IRE	irreversible electroporation
IRF	Interferon regulatory factor
ISG	IFN-stimulated genes
LNA	locked nucleic acid
m7g	7-methylguanosine
miRNA	microRNA
mRNA	messengerRNA
OAS	oligoadenylate synthetases
ORF	open reading frame
PABPC protein	poly(A)-binding protein cytoplasmatic
piRNA	PIWI-interacting RNA
PIWI	P-element induced wimpy testis
PKR	Protein kinase R
PPR	pattern recognition factor
pre-miRNA	Precursor-miRNA
pri-miRNA	Primary-miRNA
PS-ASO	Phosphorothioate-modified ASO
PTGS	post-transcriptional gene silencing
PTM	Posttranscriptional modification
rAdV	recombinant adenoviruses
RdRP	RNA-dependent RNA polymerase
RE	reversible electroporation
RISC	RNA-induced silencing complex
RNAi	RNA interference
RNAPol	RNA polymerase
RNAse	Ribonuclease
shRNA	Short hairpin RNA
siRNA	Small interfering RNA
sRNA	Small RNA
TE	transposable element
TGS	transcriptional gene silencing
TLR	Toll-like receptor
TMP	transmembrane potential
TNF-alpha	Tumor necrosis factor alpha
TRBP	Tar-RNA-binding protein
